# Status of malaria and its implications for elimination in an endemic province of South Africa: retrospective analysis

**DOI:** 10.11604/pamj.2022.41.275.26774

**Published:** 2022-04-06

**Authors:** Joyce Mahlako Tsoka-Gwegweni

**Affiliations:** 1 Faculty of Health Sciences, University of the Free State, Bloemfontein, South Africa

**Keywords:** South Africa, KwaZulu-Natal, malaria, elimination

## Abstract

South Africa set a target to eliminate malaria by 2018. Kwa-Zulu-Natal (KZN) province was earmarked to reach the elimination goal first. However, recent evidence suggests that the province has not yet achieved the elimination goal. This study set out to assess the status of malaria in KZN province of South Africa and its implications for elimination. Using retrospective analysis, the study quantified and described 5 787 cases of malaria for the years 2010-2019 in KZN province of South Africa. Data on reported malaria cases were obtained from the Malaria Information System of the KZN Provincial Department of Health. More than 65% of the cases came from male patients aged 16 years and above. A high number of cases were reported in the years 2014, 2017 and 2018. More than 40% of the cases were imported from countries outside of South Africa. The study confirms an increase in malaria cases, especially during the elimination year. The imported malaria cases and other related factors pose a threat to achieving the elimination goal. The KZN province needs to strengthen elimination efforts, including strategies to prevent imported malaria cases to achieve the new elimination goal.

## Introduction

Malaria transmission in South Africa is seasonal, occurring during the rainy summer period from September to May. The peak malaria season in South Africa takes place during the months of January to April. Currently, only 10% of the population in South Africa live in malaria transmission areas in malaria-endemic districts located in three provinces of Kwa-Zulu-Natal (KZN), Limpopo and Mpumalanga [1]. Transmission is regarded as low transmission due to the low burden of malaria in the country with the prevalence of less than 10% or incidence of 100-250 cases per 1 000 population [2]. In line with the World Health Organization (WHO) vision of ‘a world free of malaria’ [3], South Africa is among the global countries pursuing malaria elimination as well as one of the eight countries within the Southern African Development Community (SADC) aiming to eliminate malaria in the region by 2030 [4]. South Africa set a target to eliminate malaria by 2018 [5].

Until the year 2000, KZN province was historically the highest-burden malaria-endemic area and experienced severe outbreaks during 1999-2000, with cases reaching above 40 000. Through a combination of aggressive and intensive malaria control efforts, the province managed to drastically reduce the burden of malaria during 2000-2010 [6-10]. The province is now classified as a very low transmission area reporting the incidence of fewer than 0.1/1 000 people at risk [1]. The province was earmarked to reach the malaria elimination goal earlier than the other two endemic provinces of South Africa [5]. However, recent evidence suggests that the province has not been able to achieve the malaria elimination goal by 2018 due to increased malaria cases [11,12]. As a result, the elimination goal is now set for 2023 to achieve zero local malaria transmission [1]. The aim of the study was to assess the status of malaria in KZN province by quantifying and describing malaria cases for the years 2010-2019, as well as highlight some of the reported factors threatening malaria elimination in the province. Regular reporting and description of malaria cases form part of the surveillance, which is one of the important strategies for malaria elimination [2].

## Methods

**Study design and setting**: this is a descriptive study of malaria historical cases for the years 2010-2019. The study focused on the KZN province of South Africa. KwaZulu-Natal is the second most populated province in South Africa, carrying 11.3 million people and mostly rural [13]. There are now only three malaria-endemic districts in KZN, which are located near the borders of Eswatini and Mozambique [1].

**Data sources and analysis**: data on reported malaria cases for 30 November 2010 to 16 January 2019 were obtained in January 2020 from the KwaZulu-Natal Provincial Department of Health Malaria Information System (MIS) database in a Microsoft Excel spreadsheet. The MIS database is an electronic surveillance system of all malaria cases in the province [14]. Inclusion criteria consisted of only malaria case data for KZN province, reported for the years 2010-2019 and of patients of all ages and sex. A total of 5 787 records were extracted and contained information about case number, age and sex of the patient, residential locality, date of reporting the case, species of malaria parasite and travel history. The data were described as frequency values and percentages. Approval to conduct the study was granted by the University of Kwa-Zulu-Natal Biomedical Research Ethics Committee (REF BE563/17), and permission to access the database was granted by the KwaZulu-Natal Provincial Department of Health.

## Results

The total number of cases was 5 787, and 1.2% of the cases had no age recorded. In the 5 684 cases with the known age status, the range was 0-90 years. The majority of these cases were adults in the age groups 16-35 and 36-90 years. A fewer number of cases were recorded in the paediatric population. Almost two-thirds of the cases occurred in male patients, and more than 97% of the cases were due to *Plasmodium falciparum* parasite infections, while the remaining cases were shared between *Plasmodium ovale, Plasmodium vivax, Plasmodium malariae* and mixed parasite infections. Of the cases with known travel history, more than 40% had travelled outside South Africa, and the rest had travelled within malaria-endemic areas of South Africa (Table 1).

**Table 1:** characteristics of the malaria cases in KZN province

Variable	Number	Percentage
**Age** (n = 5 684)		
0-4 years	560	9.9
5-15 years	697	12.3
16-35 years	2 736	48.1
36-90 years	1 691	29.7
**Sex** (n = 5 787)		
Female	1 972	34.1
Male	3 815	65.9
**Travel history** (n = 3 735)		
Within South Africa	2 096	56.1
Outside South Africa	1 639	43.9
**Parasite** (n = 5 731)		
*Plasmodium falciparum*	5 611	97.9
Other Plasmodium parasites	103	1.8
Mixed infections	17	0.3

**Figure 1:**
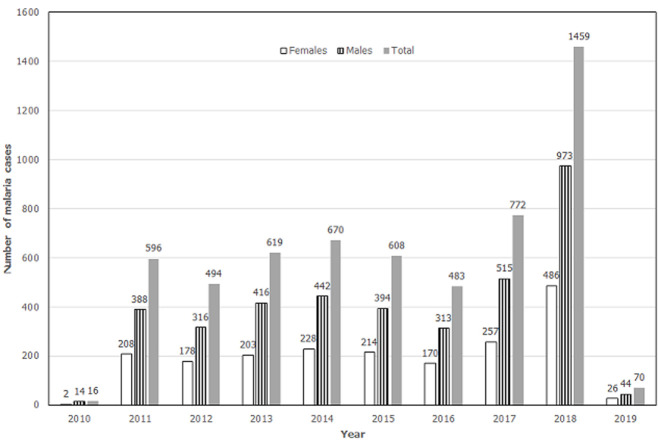
annual malaria cases in KZN province

**Figure 2:**
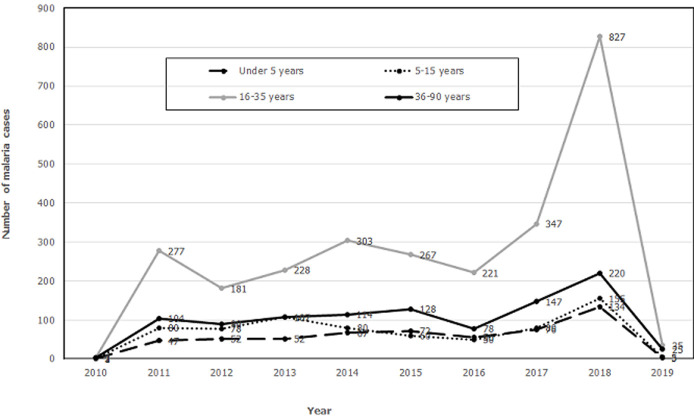
trends in age-specific malaria cases

The highest number of malaria cases were reported in the year 2018 (1 459 cases), followed by the years 2017 and 2014. The years 2013, 2015 and 2011 each recorded more than 500 cases, while the remaining years recorded below 500 cases. A similar trend was observed when disaggregating the cases by sex, even though there were more malaria cases in the males (Figure 1). The data on age-specific trends in malaria also show that cases for all age groups were highest in the year 2018. The age group most affected was 16-35 years, with the highest number of malaria cases in all the years. The other age groups had fewer cases with small peaks in the years 2011, 2014, 2015 and 2017. Malaria cases reported for the 0-4 years and 5-15 years were lower than in the adult population (Figure 2).

## Discussion

**Malaria cases**: the WHO recommends that data should be frequently collected as part of the surveillance activities towards malaria elimination [2]. The results from the present study are, therefore, important as part of the monitoring progress towards malaria elimination.

The present study shows an increase in malaria cases during the years 2014, 2017 and 2018. This finding confirms recent reports about occasional outbreaks and upsurge in malaria in the province and South Africa [1,11,14]. In South Africa, as a low transmission endemic area, all ages in the population are at risk of malaria due to the lack of immunity [15]. However, the present study found a high number of cases in the adult than in the paediatric population. This could be related to the fact that adults spend more time outside exposed to mosquitoes than children. This finding is in contrast to the high transmission areas where children, especially those under the age of five years, are more vulnerable to malaria infections [15]. The results also show that a high number of malaria cases were reported in males. This is in contrast to the picture of malaria in sub-Saharan Africa, which shows that women, especially pregnant women, are highly vulnerable to malaria infections [15]. This could be related to the fact that, globally, males are the ones that travel the most and therefore are exposed to infections.

Almost all the malaria cases were due to *P. falciparum* infections. This is expected, as *P. falciparum* remains the most common parasite transmitting malaria in sub-Saharan Africa [15]. The local transmission of cases reveals patterns of travel to malaria-endemic districts in South Africa during the transmission season, which coincides with the summer holiday period [16].

Implications for malaria elimination: many challenges have been identified as a threat to malaria elimination in the SADC region [4]. The current study highlights five of these challenges that might have contributed to the increase in malaria in KZN; imported malaria, asymptomatic infections, climatic factors, movement of people and health system factors. The present study confirms the presence of imported malaria, which has been identified as one of the threats to malaria elimination in KZN and South Africa [1,9,14,17].

Closely linked to imported cases and an increase in malaria cases in the province is the presence of asymptomatic malaria infections. Limited research has shown that asymptomatic malaria infections are present in malaria-endemic and non-endemic areas [17,18]. In their recent survey in KZN´s district known to carry a high burden of malaria, Raman *et al*. [17] reported that though the prevalence of malaria was low in the area, all the cases were asymptomatic and imported infections detected in foreign nationals. This is again worrying because asymptomatic infections remain a reservoir for local transmission and risk of re-establishment of malaria parasites into areas where conditions may promote transmission [3]. In KZN, Abiodun *et al*. [11] found that climatic factors, especially rainfall and temperature, were associated with an increased burden of malaria. This is because the same conditions were regarded as conducive for promoting the abundance of mosquito populations that transmit malaria.

Related to imported malaria and asymptomatic infections is migration and movement of people in and out of South African borders. Since the early 1990s, the South African government has allowed free entry into South Africa to enable people to seek employment opportunities, free trade and access to free government services. As a result, a high influx of migrants into South Africa has been taking place, some from high malaria-endemic countries [13]. It is reported that 817 399 people crossed KZN borders in 2018, including those coming from Mozambique and Eswatini [16]. This has been identified as a threat to malaria elimination due to the risk of imported malaria and carriage of asymptomatic infections [19]. While the present study did not particularly focus on health system factors, recent reports reveal gaps in the health system related to elimination efforts [12,14,17,20,21]. It is reported that low coverage of vector control activities may have promoted the increase in malaria-transmitting mosquito populations and resulted in the upsurge of malaria burden observed in the past three malaria seasons [12,14,17]. One of the objectives of the revised malaria elimination strategy for South Africa is to ensure 95% coverage with vector suppression interventions for all people at risk [1].

**Study limitations**: this study was limited by the following: 1) the data were collected for surveillance purposes and notifying of cases, not necessarily for research. Therefore, the data contain only those variables needed for that purpose; 2) missing data since this is a retrospective study. The author had no control over the missing information; 3) no specific sampling was applied when collecting data for malaria surveillance. The author collected all the data that were available on the cases.

## Conclusion

To conclude, the study confirms increased *P. falciparum* malaria in the KZN province, among males and young adults, particularly during the year 2018, which was set as the target year for elimination. Both the increase in malaria and imported cases, together with other related factors, pose a threat to malaria elimination. To attain the malaria elimination goal now set for 2023, it is recommended that future strategies include strengthening the health system malaria elimination efforts; and implementation of cross-border malaria interventions, such as compulsory screening to pick up both imported and asymptomatic infections. Further research is needed to provide a better understanding of the factors associated with the increase of malaria cases and other malaria preventive measures during the malaria transmission season. Efforts to prevent imported malaria cases and the feasibility of introducing cross-border malaria interventions should be investigated.

### What is known about this topic

- The burden of malaria in South Africa has declined over the past decade;
- South Africa initiated malaria elimination in 2012;
- KwaZulu-Natal province and South Africa were not able to reach the elimination goal in 2018.

### What this study adds

- The study confirms an increase in malaria cases, particularly in the year set for elimination;
- Males and young adults are the most affected by malaria;
- Imported cases of malaria remain a threat to malaria elimination in KZN province.
